# Effect of application time and concentration of silver diamine fluoride on the enamel remineralization

**DOI:** 10.4317/jced.58318

**Published:** 2021-07-01

**Authors:** Marília-Franco Punhagui, Eduardo-Inocente Jussiani, Avacir-Casanova Andrello, Jaqueline-Costa Favaro, Ricardo-Danil Guiraldo, Murilo-Baena Lopes, Sandrine-Bittencourt Berger

**Affiliations:** 1 State University of Londrina (UNOPAR), Department of Oral Medicine and Pediatric Dentistry, Londrina, PR, Brazil; 2 State University of Londrina (UEL), Department of Physics, Londrina, PR, Brazil; 3 University of North Parana (UNOPAR), Department of Restorative Dentistry, Londrina, PR, Brazil

## Abstract

**Background:**

Silver diamine fluoride has attracted attention because of its clinical success in arresting dental caries. Thus, the aim of this study was to evaluate the effect of different application times and concentrations of silver diamine fluoride (SDF) on deciduous tooth enamel remineralization.

**Material and Methods:**

Blocks of deciduous tooth enamel were categorized into six groups of 11 each: 2 control groups: intact enamel, and demineralized enamel; 38% SDF and 30% SDF which were subdivided according to application times (1 and 3 min). The microhardness of samples was determined, and all groups except the intact enamel group were subjected to pH cycling to produce initial carious lesions. The 38% and 30% SDF solutions were applied to the enamel for 1 or 3 min. After pH cycling and SDF treatments, the microhardness was again determined. Samples were sectioned to evaluate the cross-sectional microhardness. Furthermore, internal porosity of the samples was examined using micro-CT. Data were statistically analyzed by analysis of variance followed by Tukey’s test, and linear regression analyses were performed.

**Results:**

There was no difference in enamel remineralization based on surface and cross-sectional microhardness. The 30% SDF solution applied for 3 min promoted significantly less pores than the other groups.

**Conclusions:**

The 1-min application time promoted enamel remineralization regardless of the SDF concentration (30% or 38%).

** Key words:**Cariostatic agents, dental caries, primary tooth, tooth remineralization.

## Introduction

Silver diamine fluoride (SDF) is an anti-caries agent that can be used for caries prevention or as a standalone treatment in the control of dental caries, and it is applied until the child is old enough to cooperate during dental treatment. It is a non-surgical method of controlling caries (1) because the remineralization process depends on mineral changes in the hard structure of dental tissues (2). Laboratory studies demonstrate that the surface microhardness of demineralized enamel increases significantly when treated with SDF; however, a single application of a cariostatic agent elevates the surface microhardness of the demineralized enamel in the short term, and this effect of remineralization is not sustained after 7–30 days (1). SDF reapplications are therefore necessary to maintain caries prevention (3-7).

The literature identifies several inconsistencies in the clinical-related protocol for SDF application (3), where for a SDF concentration of 38% applied once a yr, the rates of caries arrest ranged from 31% to 65.6% (4,6,7) meanwhile, when applied twice a yr, the rate reached 76.3% (6). In addition, for a SDF concentration of 30% applied once a yr, rates of 40% to 79% have been reported (4,5) and when applied twice a yr, the rate increased to 91% (4). When 30% SDF was applied for 3 consecutive wk, an arrest rate of 35% was achieved (5), whereas when fluoride varnish was applied, the arrest rate ranged from 27% to 41% (4,5). Regarding cariostatic contact time, clinical studies do not report a consensus value; it can range from a minimum time of 10 s (5) and between 18 and 3 min (6,7).

Deciduous enamel is different from permanent enamel, although it presents similar prism arrangements when the prisms in deciduous enamel are smaller (9). Furthermore, deciduous teeth are characterized by lower enamel thickness (approximately 50%) than permanent teeth. They are therefore more fragile, with lesser mineralization and a negative impact on their mechanical properties. They are prone to rapid development of caries, erosion, and fractures; hence, early noninvasive treatments of incipient injuries through remineralization of the enamel surface are a constantly evolving field of research (10). These differences between deciduous and permanent enamel could also lead to different demineralization/remineralization patterns. Therefore, it is important to fully investigate the effect of SDF on deciduous enamel.

Based on the above report, this study aimed to determine if deciduous dental enamel remineralization could be influenced by SDF concentration or application time. The null hypothesis tested was that there was no difference when the deciduous enamel is treated with SDF for 1 or 3 min, regardless of the concentration.

## Material and Methods

The study was approved by the local research ethics committee (Protocol: 1.965.764). The choice of cariostatic agent was based on their concentrations, which are listed in Table 1. The intact and demineralized deciduous enamel were considered control groups.

**Table 1:**
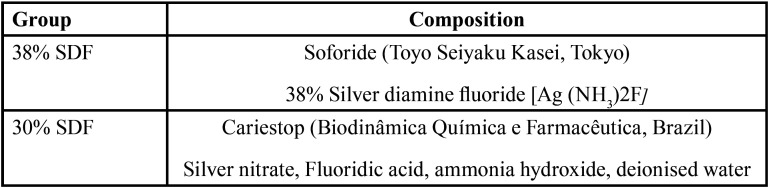
Details of the cariostatic agents used in the study, n = 11.

-Sample size calculation

The sample size of 11 enamel blocks per experimental group was calculated based on a pilot study, considering the surface microhardness as the primary outcome (recovery of microhardness from demineralized enamel after treatment). A microhardness recovery difference of 80 Knoop hardness number (KHN) with a standard deviation of 42 KHN, an α-error of 0.05, and a power (1-ß) of 0.9 were used.

-Sample preparation

We included 45 healthy, human, naturally erupted, and exfoliated deciduous molars that had been stored in 0.5% chloramine T at 4 °C. The molars were utilized within 1 month. The teeth were cleaned with periodontal curettes, and prophylaxis was performed with pumice stones, with the aid of a Robinson brush for complete removal of debris. The roots of all the teeth were sectioned using a double-edged diamond disc (KG Sorensen, Barueri, SP, Brazil) at a location 2 mm above the dentin–enamel junction. Next, 90 enamel blocks were obtained (4 × 4 × 3 mm) from buccal and lingual faces using a double-sided diamond disc (KG Sorensen, Barueri, SP, Brazil) (11).

The blocks were fixed on acrylic discs, with the dentin face in contact with the disc and the enamel surface exposed parallel to the disc surface, and then fixed with sticky wax. The enamel surface was abraded with silicon carbide on granulations of 1000 (for 20 s) and 1200 (for 40 s) at low rotation on the polisher (APL4, Arotec, Cotia, SP, Brazil) for surface planning and subsequently polished with diamond paste and felt discs of 1 and 1/4 µm (Arotec, Cotia, SP, Brazil). The samples were placed in an ultrasonic vat with deionized water (Unique Indústria e Comércio de Produtos Eletrônicos, São Paulo, SP, Brazil) for 10 min to remove waste. The samples were then isolated with red nail polish to delimit an area of 7 mm² exposure.

Initial surface microhardness (SHi) and sample selection

The samples were maintained with the enamel surface (test surface) parallel to the acrylic base. The SH test was performed to give three impressions 100-μm apart on the central area of the block with a Knoop-type penetrator (HMV-G, Shimadzu, Tokyo, Japan) and a static charge of 25 g for 5 s. The overall mean microhardness of the 120 enamel blocks was calculated (KHN = 357.36 ± 35.73), and the values above and below 10% of the average were excluded from the study; sample homogeneity was verified using one-way analysis of variance (ANOVA) (*P* > 0.05). The 66 samples were then numbered and randomly allocated according to a list generated by RANDOM.ORG and divided into six experimental groups with 11 samples per group.

-pH-cycling to obtain initial caries lesion

All enamel samples, except those destined to remain intact, were subjected to pH cycling with demineralizing and remineralizing solutions at 37 °C. The samples were immersed for 16 h in a demineralizing solution (0.05 mol/L acetate buffer containing 1.28 mmol/L Ca, 0.74 mmol/L P, and 0.03 µg/mL F; pH 5.0) and for 8 h in a remineralizing solution (0.1 mol/L Tris buffer containing 1.5 mmol/L Ca, 0.9 mmol/L P, 150 mmol/L KCl, and 0.05 µg/mL; pH 7.0) at 37°C. Microhardness was determined daily for one sample from each group until a KHN value close to 150 was obtained (11).

-SH after carious lesion (SHpH)

The 1- and 3-min samples from the demineralized enamel, 38% SDF, and 30% SDF groups were again subjected to the microhardness test to verify enamel demineralization through three impressions 100 μm apart on the central area of the block with a Knoop penetrator (HMV-G) and a static charge of 25 g for 5 s. Then, the mean microhardness of each sample was calculated.

-Cariostatic application

After the SH test (SHpH), prophylaxis with water and pumice stone was performed using a Robinson toothbrush. Then, the enamel surfaces were washed and dried. An applicator was dipped into the cariostatic agent (38% SDF or 30% SDF), and 3-4 mg was applied for 1 or 3 min to the enamel surface (one drop was used for three samples).

-SH after cariostatic agent application (SHf)

After 24 h, the treated samples were again subjected to the SH test to verify enamel remineralization, followed by calculation of the mean. The SHi, SHpH, and SHf values were used to calculate the percentage of surface remineralization (%SH) using the following formula.

%SH = (SHf − SHpH) / (SHi − SHpH) × 100

The difference in microhardness (ΔSH) was obtained by subtracting SHf from SHpH (12). The percentage of remineralization was decreased in the demineralized enamel samples because these samples had been subjected to caries lesion *in vitro* (pH cycling) but had not been subjected to cariostatic agent application. In the intact enamel samples, there was no change in the percentage of remineralization when the blocks were not subjected to pH cycling and cariostatic agent application.

-Cross-sectional microhardness

For determination of the internal cross-sectional microhardness of the samples and in-depth evaluation of the effect of the cariostatic agent on the dental enamel, the specimens were sectioned on their long axis using a diamond disc in-precision cutter (Isomet 1000, Buehler, Lake Bluff, IL, USA). The inner surface was abraded using silicon carbide sandpaper (granulations of 1.000 and 1.200) and polished with felt discs and diamond paste (granulations of 1 and 1/4 µm). Three impressions of internal microhardness were measured, with the following distances from surface: 10, 20, 50, 70, and 90 µm (13).

-Micro-CT evaluation - internal porosity

For micro-CT evaluation of the superficial and internal porosity of the deciduous dental enamel, four representative samples from each group were used. The samples were scanned using a Bruker SkyScan, model 1172 (Bruker BioSpin Corporation, Kontich, Belgium). The scans were performed in stresses in the range of 70–90 kV and a sample-dependent resolution of 4 to 8 µm. All measurements were performed using an aluminum filter with an angular pitch of 0.4° between the projections and rotated at 180°. To determine the superficial and internal porosity of the samples, the images were reconstructed and analyzed using NRecon, DataView, and CTAn software together with micro-CT (14).

-Data analysis

The data were tabulated and assessed for normality and homoscedasticity using the Kolmogorov–Smirnov and Bartlett’s tests, respectively, in Minitab 16 for Windows 8 software (Minitab, Pennsylvania State College, Philadelphia, PA, USA). The data presented normal distribution and homoscedasticity.

Data from SH and cross-sectional microhardness were subjected to three-way ANOVA followed by Tukey’s test considering the following factors: cariostatic agent, application time (1 or 3 min) and assessment time (SHi, SHpH and SHf) of SH, and depth of cross-sectional microhardness. In addition, %SH, ΔSH, and micro-CT data were analyzed using one-way ANOVA followed by Tukey’s test. Dunnett’s test was used to compare micro-CT data between the experimental and control groups (demineralized and intact enamel groups). For all analyses, an α threshold value of 0.05 was used to determine statistical significance.

## Results

-SH and cross-sectional microhardness

The ANOVA results of SH did not identify any difference among the SDF concentrations (30% or 38% SDF; *P* = 0.853), but differences in application time (1 or 3 min, *P* < 0.001) and assessment time (SHi, SHpH, and SHf, *P* = 0.003) were observed. There was no significant difference in %SH and ΔSH (*P* = 0.465 and *P* = 0.381, respectively). Table 2 shows the SH values according to SDF application time and concentration. There was a statistically significant difference among the assessment times (SHi > SHpH < SHf; *P* = 0.003). The %SH and ΔSH were statistically similar for all groups. Additionally, linear regression analysis demonstrated that 4% ΔSH (adjusted R-squared) is explained by SDF concentration and application time.

**Table 2:**
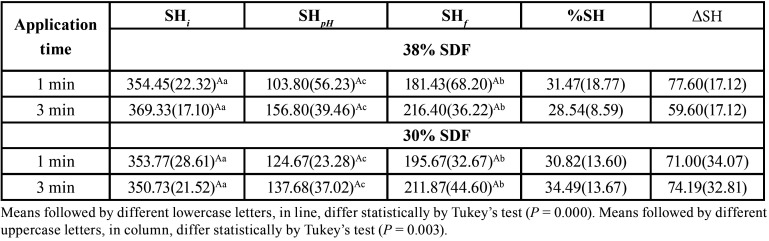
Mean values (SD) of the initial surface microhardness (SHi), after pH-cycling microhardness (SHpH), final surface microhardness (SHf), percentage of surface remineralization (%SH) and variation of microhardness (ΔSH), n = 11.

For cross-sectional microhardness, ANOVA results showed a statistically significant difference for enamel treatment (intact enamel, demineralized enamel, 30% SDF, and 38% SDF, *P* < 0.001) and depths (10, 30, 50, 70 and 90 µm, *P* = 0.025). Table 3 shows that demineralized enamel had significantly lower cross-sectional microhardness than intact enamel and enamel treated with 30% and 38% SDF. Furthermore, there was no significant difference between the SDF concentrations. On analyzing the depths, only a difference between 10 and 90 µm was noted, regardless of the enamel treatment. The linear regression analysis demonstrated that 38% cross-sectional microhardness (adjusted R-squared) was explained by enamel treatment, depth, and application time.

**Table 3:**
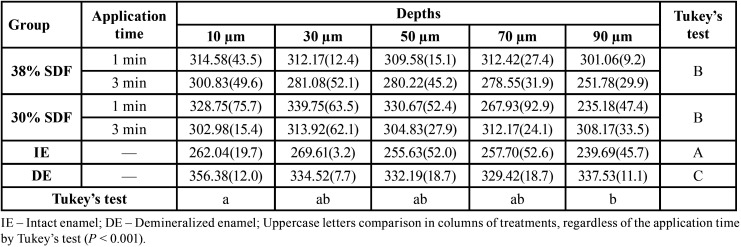
Mean values (SD) of cross-sectional microhardness according to the treatments, application time and depth, n = 11.

-Micro-CT - internal porosity

In the micro-CT data, ANOVA results identified a significant difference between the factors (enamel treatment and application time, *P* < 0.001) as well as an interaction between them (enamel treatment vs. application time, *P*< 0.001). Table 4 shows the means values of internal porosity; it can be observed that treatment with 30% SDF led to the lowest internal porosity. The internal porosity of demineralized enamel was statically similar to that of enamel treated with 38% SDF, independent of the application time. Moreover, only treatment with 30% SDF for 3 min was similar to intact enamel. The linear regression analysis demonstrated that 14% of internal porosity (adjusted R-squared) was explained by enamel treatment and application time.

**Table 4:**
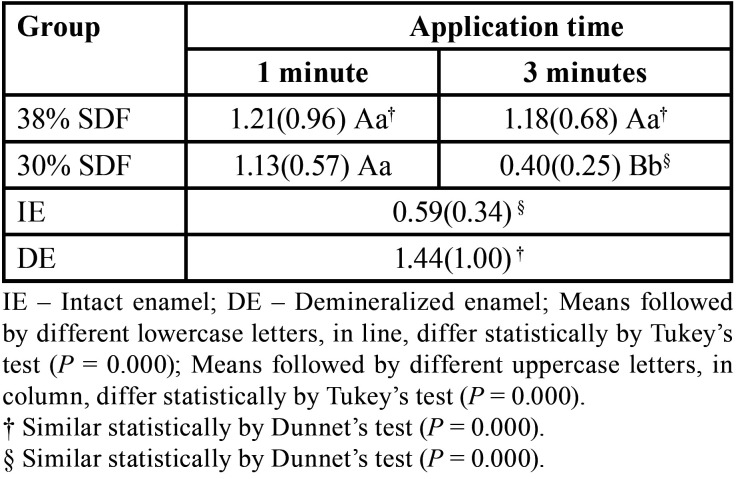
Values of internal porosity values (standard deviation) obtained through micro-CT.

## Discussion

This study evaluated the effect of SDF application time (1 and 3 min) and concentration (30% and 38%) on the remineralization of deciduous dental enamel. Based on the results, the null hypothesis was rejected because, in general, there was no difference in the remineralization of the surface independent of the SDF application time. 

SDF, which is composed of fluoride and silver ions, acts by blocking demineralization, which is associated with its antibacterial properties.14 Godoi *et al*. (15) reported that artificially-induced carious lesions showed increased mineral density after SDF application, which is in line with the findings of the present study. As the results indicate, the superficial remineralization in deciduous enamel treated with SDF is independent of SDF application time or concentration (Table 2). The results show that the %SH increased from 28.54% to 34.49%, with evident remineralization following both 1 and 3 min of contact with cariostatic agents, but without a significant difference between SDF application times and concentration. These findings are similar to those of Scarpelli *et al*. (16) but are different from those of Punyanirun *et al*. (17), who reported a higher percentage of remineralization using SDF (42.56%). This difference in results could be explained by differences in the substrate used and the type of enamel: deciduous16 or permanent (17).

In vitro studies reported a decrease in deep demineralization in the enamel lesion after SDF application (18,19). The results presented in our study also report this fact in the cross-sectional microhardness test, as shown in Table 2; cross-sectional microhardness decreased with depth. It was expected that longer application time would promote more in-depth SDF penetration. In a study using an in situ model (19), it was observed that SDF had significantly higher microhardness than that in the control group (without treatment) at a depth of 0–84 μm. This may be because an in situ model represents possible exposure to the oral environment, wherein the substrate is subjected to the complexity of local microbiota and the dynamic balance of demineralization and remineralization.

Micro-CT is a noninvasive technique capable of detecting carious lesions and remineralization zones *in vitro*, thereby allowing the evaluation of superficial and internal porosity of deciduous enamel (14). The micro-CT results indicate that 30% SDF applied for 3 min promoted fewer pores, which corresponds to greater enamel mineralization. These results contradict the findings of Gao *et al*. (20), who in a systematic review reported that 38% SDF is more effective than SDF in lower concentrations. This phenomenon may have occurred because of the composition of this product, and the solvent could have promoted a higher penetration. Furthermore, a previous study that used micro-CT and evaluated mineral density reported a significant mineral density gain using SDF, ranging from 0 to 260 μm. These contradictory results could be explained by the difference in data processing. The present study quantified pores within the samples; additionally, the samples were thin due to the limited thickness of the substrate.

The concentration of the cariostatic agent evaluated in this study does not refer to the fluoride and silver ion concentrations but to the final concentration found in the commercial product (21). However, regarding the efficacy of SDF, factors such as the concentration of fluoride and silver ions are very important. According to Chibinski *et al*. (22), high concentrations of 38% SDF (44,800 ppm F; 253,870 ppm Ag) (23) and 30% SDF (35,400 ppm F; 200,400 ppm Ag)5 provided greater efficacy in the prevention of dental caries in deciduous teeth than low concentrations (7,24). The results of this study show that even with differences in product concentration, surface enamel remineralization occurred in human deciduous teeth *in vitro* because for all analyses performed in this study (SH, cross-sectional microhardness, and micro-CT), the linear regression revealed no effect of the SDF application time or concentration on the results.

This study has certain limitations. Deciduous enamel is thicker than permanent enamel (9,10). This was observed in the present study, and it was necessary to adapt the pH cycling model to obtain carious lesions in the deciduous tooth enamel, producing similar clinical cariogenic conditions, in which the demineralizing action exceeded the remineralizing action (11,13). It is important to note that most studies evaluate the effect of cariostatic agents on permanent enamel, and this fact limits the correlation among studies. Furthermore, the absence of *in vivo* conditions such as salivary enzyme attacks, continuous changes in pH, and oral cavity temperature might mitigate the effects on enamel remineralization.

## Conclusions

On the basis of the results obtained in this study, it can be concluded that enamel remineralization occurs regardless of the application time and concentration of SDF.
